# Supplementary material for: Interface modes in planar one-dimensional magnonic crystals
Source: Sci Rep. 2022 Jul 5;12:11335. doi: 10.1038/s41598-022-15328-x (PMC9256740; doi:10.1038/s41598-022-15328-x)
Supplement: Supplementary file 1 — Supplementary Information. [file 41598_2022_15328_MOESM1_ESM.pdf]

Interface modes in planar one-dimensional magnonic crystals: Supplementary information

Szymon Mieszczak[1,*] **and Jarosław W. Kłos**[1,+]

[1]Institute of Spintronics and Quantum Information, Faculty of Physics, Adam Mickiewicz University, Poznań, Poland
[*]szymon.mieszczak@amu.edu.pl

ABSTRACT

We present the concept of Zak phase for spin waves in planar magnonic crystals and discuss the existence condition of interface modes localized on the boundary between two magnonic crystals with centrosymmetric unit cells. Using the symmetry criterion and analyzing the logarithmic derivative of the Bloch function, we study the interface modes and demonstrate the bulk-to-edge correspondence. Our theoretical results are verified numerically and extended to the case in which one of the magnonic crystals has a non-centrosymmetric unit cells. We show that by shifting the unit cell, the interface modes can traverse between the band gap edges. Our work also investigate the role of the dipolar interaction, by comparison the systems both with exchange interaction only and combined dipolar-exchange interactions.

1 Zak phase for spin waves in planar magnonic crystals

Exchange spin waves

The linearized Landau-Lifshitz-Gilbert equation can be written in the following form when the dipolar interactions are neglected $\boldsymbol{H}_{\mathrm{dm}} = 0$:

$$\begin{cases} \partial_t m_{\parallel}(x,t) = |\gamma|\mu_0 \left(\hat{L}(x) m_{\perp}(x,t) - V m_{\perp}(x,t)\right) \\ \partial_t m_{\perp}(x,t) = |\gamma|\mu_0 \left(-\hat{L}(x) m_{\parallel}(x,t) + V m_{\parallel}(x,t)\right), \end{cases} \tag{1}$$

where the function $V(x)$ and operator $\hat{L}(x)$ are defined as:

$$\begin{cases} V(x) = \left(\partial_x \lambda^2 \partial_x M_{\mathrm{S}}(x)\right) + H_0 \approx H_0 \\ \hat{L}(x) = M_{\mathrm{S}}(x) \partial_x \lambda^2(x) \partial_x \end{cases} \tag{2}$$

and λ is exchange length. We neglect the static term $\partial_x \lambda^2 \partial_x M_{\mathrm{S}}(x)$ because it is nonzero only at the interface between strips and can be neglected in numerical computations[1]. To find the eigenmodes, we are considering the harmonic dynamics in time: $\boldsymbol{m}(x,t) = \boldsymbol{m}(x) e^{i\omega t}$. The equation (1) is a Sturm-Liouville problem, can be written in the form analogous to Schrödinger equation:

$$\partial_t |m\rangle = \Lambda |m\rangle, \tag{3}$$

where we used the notation $|m\rangle := [m_{\parallel}(x), m_{\perp}(x)]^T e^{i\omega t}$ and the matrix Λ is defined as following:

$$\Lambda = |\gamma|\mu_0 \begin{pmatrix} 0 & -V + \hat{L} \\ V - \hat{L} & 0 \end{pmatrix}. \tag{4}$$

Let us consider the continuous transition of the vector $|m\rangle$ in the momentum space of the wavenumber k, after which it acquires the phase $\varphi(t)$:

$$|m'\rangle = e^{i\varphi} |m\rangle. \tag{5}$$

Eq. (3) is satisfied for $|m'\rangle$ as well. We can write the following relation resulting from (3):

$$\langle m|\partial_t|m'\rangle = \langle m|\Lambda|m'\rangle, \tag{6}$$

where we defined the inner product $\langle f_1 \mid f_2 \rangle := \int_{-a/2}^{a/2} \left(f_{1,\parallel}^*(x) f_{2,\parallel}(x) + f_{1,\perp}^*(x) f_{2,\perp}(x) \right) dx$. By differentiating $|m'\rangle$ in time and using the identity: $\partial_k = \dot{k}\partial_t$, we can write (6) in the form:

$$i\dot{\varphi} \langle m|m \rangle + \langle m|\partial_k|m \rangle \dot{k} = i\omega \langle m|m \rangle . \tag{7}$$

Taking into account that $|u\rangle = |m\rangle e^{-ikx} e^{-i\omega t}$, we obtain form (7):

$$\varphi = \underbrace{\int_o^t \omega(t)\, dt}_{\varphi_t} + \underbrace{\int_{k(0)}^{k(t)} \frac{i \langle u|\partial_k|u\rangle}{\langle u|u\rangle} dk}_{\varphi_g} . \tag{8}$$

The phase φ contains an additional term φ_g which is distinguishable from the phase φ_t acquired from the temporal evolution of the eigenmode $|m\rangle$. The geometrical phase φ_g collected when Bloch function $|m\rangle$ passes the periodic path in the space of k-number (i.e., when k is real and ranges from -π/a to π/a) is called Zak phase θ:

$$\theta = \int_{-\pi/a}^{\pi/a} \frac{\Im \langle u|\partial_k|u\rangle}{\langle u|u\rangle} dk. \tag{9}$$

Dipolar-exchange spin waves

The demagnetizing field $\boldsymbol{H}_{\mathrm{dm}}(x,z,t) = -\nabla\varphi(x,z,t)$ is calculated under magnetostatic approximation by finding the magnetostatic potential $\varphi(x,z,t)$ from the Gauss equation. For the layer which is periodically modulated in the plane, the demagnetizing field was calculated using the method proposed by J. Kaczer[2], which is based on the Fourier expansion. For thin planar structure, the demagnetizing field does not change significantly inside the magnetic layer and we took its value in the middle of the layer ($z = 0$) as a representative for the whole cross-section of the layer. The dynamic demagnetizing field is expressed in terms of the coefficients of the Fourier expansion of the Bloch function $|m\rangle$, therefore the Eq. (3) must be written in the Fourier space as well:

$$\partial_t |\tilde{m}\rangle = \tilde{\Lambda} |\tilde{m}\rangle \tag{10}$$

where $|\tilde{m}\rangle := [u_{\parallel,G_0}, \ldots, u_{\parallel,G_n}, \ldots, u_{\perp,G_0}, \ldots, u_{\perp,G_n}, \ldots]^T e^{ikx} e^{i\omega t}$ with $u_{\parallel,G_i}$, $u_{\perp,G_i}$ being the Fourier coefficients, and matrix $\tilde{\Lambda}$ taking a form:

$$\tilde{\Lambda} = |\gamma|\mu_0 \begin{pmatrix} 0 & -\tilde{V} + \tilde{L} + \tilde{D}^{\parallel,\perp} \\ \tilde{V} - \tilde{L} - \tilde{D}^{\perp,\parallel} & 0 \end{pmatrix}. \tag{11}$$

The matrices $\tilde{L}$ and $\tilde{V}$ are related to (2) and describe the impact of external field and exchange interactions, respectively. The matrices $\tilde{D}^{\perp,\parallel}$ and $\tilde{D}^{\parallel,\perp}$ describe the dynamic dipolar interactions in 1D planar magnonic crystal[1,2] – the difference between them is reflected in the ellipticity of pressecion of dipolar spin waves. The explicit form of these matrices is:

$$\begin{aligned} \tilde{L}_{i,j} &= -\sum_l (k+G_i)(k+G_l)\lambda^2_{G_l-G_j} M_{S,G_i-G_l}, \\ \tilde{V}_{i,j} &= H_0 \delta_{i,j}, \\ \tilde{D}^{\perp,\parallel,}_{i,j} &= -\left(1 - e^{-|k+G_j|d/2}\right) M_{S,G_i-G_j}, \\ \tilde{D}^{\parallel,\perp}_{i,j} &= -e^{-|k+G_j|d/2} M_{S,G_i-G_j}, \end{aligned} \tag{12}$$

where d is the thickness of the layer. The material parameters, i.e., saturation magnetization $M_S(x)$, exchange length $\lambda(x)$ and the components of Bloch function: $m_\parallel(x)$, $m_\perp(x)$ are expanded into Fourier series:

$$\begin{aligned} M_S(x) &= \sum_{n=0} M_{S,G_n} e^{iG_n x}, \\ \lambda(x) &= \sum_{n=0} \lambda_{G_i} e^{iG_n x}, \\ m_{\parallel(\perp)}(x) &= \sum_{n=0} u_{\parallel(\perp),G_n} e^{i(G_n+k)x}. \end{aligned} \tag{13}$$

The set $\{G_n\} = 0, \pm 2\pi/a, \pm 4\pi/a, \ldots, \pm n2\pi/a, \ldots$ denotes the reciprocal lattice numbers.

To prove that the formula (9) also applies to dipolar-exchange spin waves, we need to show that $\langle u|\partial_k|u\rangle = \langle\tilde{u}|\partial_k|\tilde{u}\rangle$ and $\langle u|u\rangle = \langle\tilde{u}|\tilde{u}\rangle$, where $|\tilde{u}\rangle = |\tilde{m}\rangle\, e^{-ikx}e^{-i\omega t}$:

$$\langle u|\partial_k|u\rangle = \int_{-a/2}^{a/2}\left(u_{\|}^{*}(x)\partial_k u_{\|}(x) + u_{\perp}^{*}(x)\partial_k u_{\perp}(x)\right)dx =$$
$$\sum_{i,j}\underbrace{\int_{-a/2}^{a/2} e^{i(G_i-G_j)x}dx}_{\delta_{i,j}}\left(u_{\|,G_i}^{*}\partial_k u_{\|,G_j} + u_{\perp,G_i}^{*}\partial_k u_{\perp,G_j}\right) = \sum_i\left(u_{\|,G_i}^{*}\partial_k u_{\|,G_i} + u_{\perp,G_i}^{*}\partial_k u_{\perp,G_i}\right) =: \langle\tilde{u}|\partial_k|\tilde{u}\rangle\,. \tag{14}$$

The relation $\langle u|u\rangle = \langle\tilde{u}|\tilde{u}\rangle$ can be proven in the same way. Starting from (10), we can then show that the Zak phase for dipolar-exchange spin waves is also equal:

$$\theta = \int_{-\pi/a}^{\pi/a}\frac{\Im\,\langle\tilde{u}|\partial_k|\tilde{u}\rangle}{\langle\tilde{u}|\tilde{u}\rangle}dk = \int_{-\pi/a}^{\pi/a}\frac{\Im\,\langle u|\partial_k|u\rangle}{\langle u|u\rangle}dk. \tag{15}$$

In (13, 14) we omitted the indexing of $u_{\|(\perp)}(x)$ and their Fourier coefficients $u_{\|(\perp),G_n}$ by the band number and did not marked their dependence on the wavenumber k.

2 Zak phase and logarithmic derivative for the crystal with centrosymmetric unit cells

For 1D crystal the Bloch function at $k = 0$ or $k = \pm\pi/a$ is periodic ($\boldsymbol{m}(x+a) = \boldsymbol{m}(x)$) or anti-periodic ($\boldsymbol{m}(x+a) = -\boldsymbol{m}(x)$), respectively. Moreover, for the crystals with centrosymmetric unit cells, the Bloch function are even ($\boldsymbol{m}(x_s+x) = \boldsymbol{m}(x_s-x) \Rightarrow \partial_x\boldsymbol{m} = 0|_{x=x_s}$) or odd ($\boldsymbol{m}(x_s+x) = -\boldsymbol{m}(x_s-x) \Rightarrow \boldsymbol{m} = 0|_{x=x_s}$) function, with respect to each of two symmetry centers $x_s = na$ or $x_s = a/2+na$, and can be normalized to be real-valued. This gives four possible type of bands, by considering the (even or odd) symmetry of the Bloch function on each of two edges of the band. It is worth noting, that the symmetry of Bloch function is the same at both symmetry centers ($x_s = 0+na$ and $x_s = a/2+na$) only for $k = 0$ whereas it is reversed (from even to odd or from odd to even) when we change the symmetry point, for $k = \pi/a$. Therefore, it is better to use Wannier functions, defined as:

$$\boldsymbol{a}(x-na) = \sqrt{\frac{a}{2\pi}}\int_{-\pi/a}^{\pi/a}\boldsymbol{m}_k(x)e^{-ikna}dk, \tag{16}$$

to classify the symmetry of the bands[3,4]. The Wannier functions characterize whole band (do not depend on the wavenumber k). For the case of crystal of centrosymmetric unit cells, they are exponentially localized around one of symmetry centers ($x_c = 0$ and $x_c = a/2$), and are either even or odd with respect to this symmetry center. The periodicity (and anti-periodicity) and symmetry of the Bloch function at $k = 0$ ($k = \pi/a$) can be strictly connected to the properties of the Wannier functions by the relation:

$$\boldsymbol{m}_k(x) = \sqrt{\frac{a}{2\pi}}\sum_{n=-\infty}^{\infty}\boldsymbol{a}(x-na)e^{ikna}, \tag{17}$$

which can be used to express the Zak phase in terms of Wannier functions. Assuming that the Bloch functions are normalized $\langle m|m\rangle = 2\pi/a \Leftrightarrow \langle a|a\rangle = 2\pi/a$, we can obtain from (15) and (17):

$$\begin{aligned}\theta &= \frac{2\pi}{a}\int_{-\pi/a}^{\pi/a} i\,\langle u|\partial_k|u\rangle\,dk \\ &= \int_{-a/2}^{a/2}\Big(\sum_{n,n'=-\infty}^{\infty}(x-na)\,\boldsymbol{a}^{*}(x-n'a)\cdot\boldsymbol{a}(x-na)\underbrace{\int_{-\pi/a}^{\pi/a}e^{ik(n-n')a}dk}_{2\pi/a\,\delta_{n,n'}}\Big)dx \\ &= \frac{2\pi}{a}\int_{-\infty}^{\infty}x|\boldsymbol{a}(x)|^2dx = \frac{2\pi}{a}\int_{-\infty}^{\infty}x(|a_{\|}(x)|^2+|a_{\perp}(x)|^2)dx,\end{aligned} \tag{18}$$

where $a_{\|}(x)$ and $a_{\perp}(x)$ denote the components of Wannier function corresponding to in-plane and out-of-plane components of Bloch function. For the system with centrosymmetric unit cells, the integral $\theta = \frac{2\pi}{a}\int_{-\infty}^{\infty}x|\boldsymbol{a}(x)|^2dx$ takes only two possible values 0 and π which correspond to different symmetry center: ($x_c = 0$ or $x_c = a/2$) at which the Wannier function, related to given band, is localized. This allows spliting all band to two disjoint topological classes where the Zak phase θ is equal to 0

or π. Please note that in general case, i.e., when the unit cells are not centrosymmetric then the Zak phase can take arbitrary value. To prove the quantized values of Zak phase for centrosymmetric unit cell, we need to discuss the symmetry of function $x|\boldsymbol{a}(x)|^2$. This function is odd when $x_c = 0$, regardless if $\boldsymbol{a}(x)$ is even or odd, which gives $\theta = 0$. For $x_c = a/2$, we need make a substitution $x \to x + a/2$ for the variable inside the integral. Then, we can find that the expression can be split into two terms: $(x+a/2)|\boldsymbol{a}(x+a/2)|^2 = x|\boldsymbol{a}(x+a/2)|^2 + a/2|\boldsymbol{a}(x+a/2)|^2$, where the first one is odd and second one is even and does not vanish after integration, which gives $\theta = \pi$. The above discussion relates the symmetry of the Bloch function on the edges of the band to the Zak phase for this band in the structures with centrosymmetric unit cells. *If the symmetry of Bloch function, respect to the center of unit cell, is the same on both edges of the band then the Zak phase for this band is equal to* 0*, otherwise to* π*.* As we noticed at beginning of Section 2, the centering of the unit cell at alternative symmetry center x_s (i.e., shifting it by $a/2$) changes the symmetry of the Bloch function on one edge of the band only, i.e., for the edge at which $k = \pi/a$. As a result *the shift of the centrosymmetric unit cell by the half of the period:* $x_s \to x_s + a/2$ *flips the Zak phase for each band:* $\theta \to \theta + \pi$.

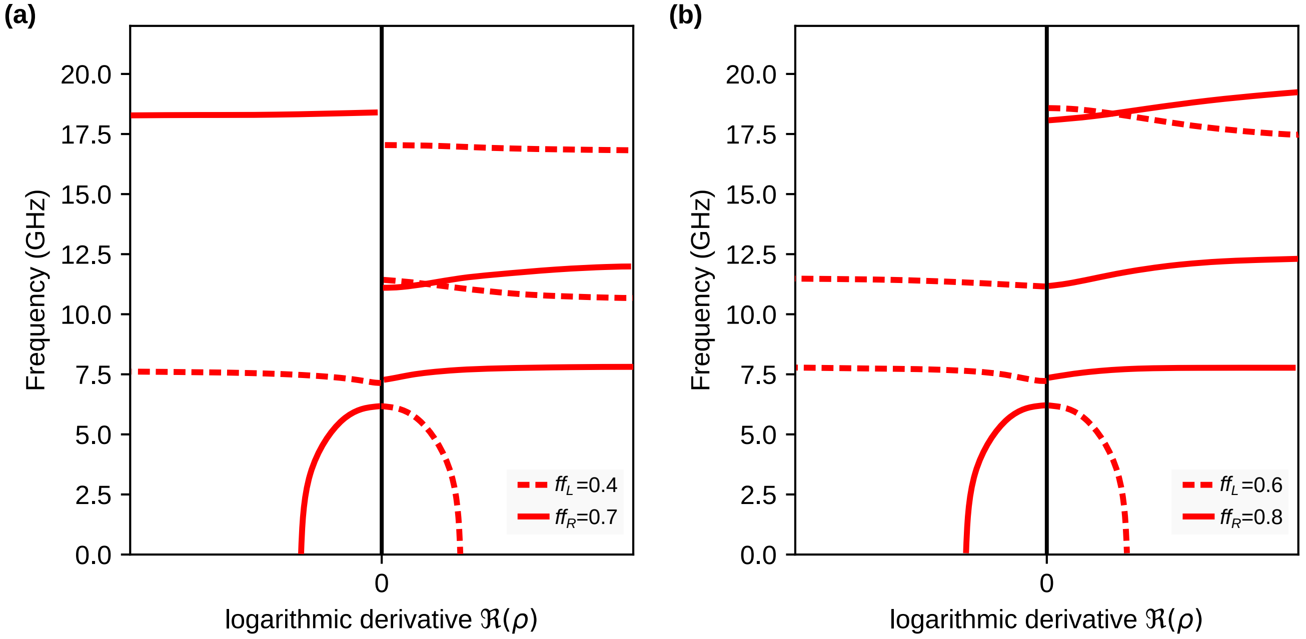

Figure 1. Logarithmic derivative calculated numerically from eq. (19). The lines (dashed for $ff = 0.4$, solid for $ff = 0.7$) are monotonically goes between 0 and infinity within the band gap. The crossing point for left and right magnonic crystals (MCs) point out the frequency value of the interface state. Here, the interface state appears only in the second band gap. Logarithmic derivative calculated numerically from eq. (19). The lines (dashed for $ff = 0.6$, solid for $ff = 0.8$) are monotonically goes between 0 and infinity within the band gap. The crossing point for left and right MCs point out the frequency value of the interface state. Here, the interface state appears only in the third band gap.

For the crystal with centrosymmetric unit cells, the properties of the Bloch function on the edges of bands are decisive for the sign of the logarithmic derivative of Bloch functions inside the gaps and thus determine the conditions of existence for surface/interface modes. According to the work of J. Zak[5]: (i) the logarithmic derivative $\rho(k)$, taken in symmetry points $x_s = na$ or $x_s = a/2 + na$, is real and has a constant sign in the whole range of the frequency gap, whereas in the band $\rho(k)$ is purely imaginary; (ii) the sign of $\rho(k)$ in two successive gaps is different (the same) if $\rho(k)$ reaches two zeros or two poles (one zero and one pole) at the edges of the band between the gaps. The zeros and poles of $\rho(k)$ at the edges of bands correspond to $\boldsymbol{m}_k|_{x=x_0} = 0$ and $\partial_x \boldsymbol{m}_k|_{x=x_0} = 0$, which means the odd and even Bloch functions at symmetry point corresponding to the edge of unit cell, respectively; (iii) the sign of ρ is conserved (negated) in the direct gaps which is opened at $k_r = 0$ ($k_r = \pi/a$), in the center of the BZ; (iv) the change of the side of interface $x_{0+} \to x_{0-}$ and the direction of decaying of the mode from $x \to \infty$ to $x \to -\infty$ (when we switch from the MC on the right to the MC on the left) requires the change of the sign of imaginary part of the wavevector from $-k_i$ to k_i, that results in the change of the sign of the logarithmic derivative: $\rho(k_r + ik_i)|_{x=x_{0+}} = -\rho(k_r - ik_i)|_{x=x_{0-}}$. The properties (i), (ii) and conclusions highlighted at the end of the previous paragraph allow us to formulate the following statement. *When the Zak phase for a given band is equal* 0 *(*π*) then the signs of logarithmic derivatives of Bloch function in gaps surrounding this band are the same (are opposite).*

The knowledge of the Zak phases for all bands below a given gap allows determining the sign of the logarithmic derivative

of Bloch function $\rho(k)$ in this gap. To justify this formula, let us start with the discussion concerning the sign of $\rho(k)$ below the lowest band $n = 1$. For the frequencies below the 1st band, the Bloch function has a form of evanescent wave ($|k_i| > 0$) with homogeneous phase ($k_r = 0$) and without the oscillations within the unit cells. Therefore, it decays monotonously to the left $x \to -\infty$ ($k_i > 0$) or to the right $x \to \infty$ ($k_i > 0$) and its logarithmic derivative is positive or negative, respectively. This property is included by the sign $\pm$ in the formula which links the sign of $\rho(k)$ in the gap with the sequence of Zak phases for the bands below it (Eq.(5) in the manuscript). When all bands are characterized by the Zak phase $\theta = 0$, the logarithmic derivative of Bloch function ρ flips its sign from the gap to the gap. Therefore, in the gap just above n^{th} band, it will be equal to $\pm(-1)^m$. The number of flips of the sign will be reduced if some bands with the Zak phase $\theta = \pi$ appear below the selected gap. If the odd (even) number of such bands exit, then the sign $\pm(-1)^m$ will (will not) be reversed. This potential reversal of the sign can be described by adding factor $\prod_{m=1}^{n} \exp(i\theta_m) = \exp\sum_{m=1}^{n}(i\theta_m)$, where the expression $\exp(i\theta_m)$ is equal to $+1$ or -1, if $\theta_m = 0$ or π, respectively.

3 Calculations of the logarithmic derivative of Bloch function using the PWM

Using the Fourier expansion of the Bloch function (13), we can strictly calculate the derivative $\partial_x m_{\parallel(\perp)}$ and write the logarithmic derivative of Bloch function in the form:

$$\rho(x) = i\frac{\sum_i (k+G_i) u_{\parallel(\perp),k,G_i} \exp(iG_i x)}{\sum_n u_{\parallel(\perp),k,G_i} \exp(iG_i x)}. \tag{19}$$

4 The exemplary SWs' profiles on the edges of the frequency bands

For the centrosymmetric unit cell, the Zak phase θ_n of each nth band of the 1D crystal can be determined only by inspection of the Bloch functions' symmetry $m_{k,\parallel(\perp),n}(x)$ in the symmetry point of the 1st Brillouin zone: $k = 0$ or $k = \pm\pi/a$, where a is a period of 1D lattice. The Zak phase of the nth band is 0 if either $|m_{n,k=0}(x = x_0)| = |m_{n,k=\pi/a}(x = x_0)| = 0$ or $|\partial_x m_{n,k=0}(x = x_0)| = |\partial_x m_{n,k=\pi/a}(x = x_0)| = 0$, where x_0 denotes the center of the unit cell. Otherwise, it is π[6].

Figs. 2(a, b) presents the profiles of modes for four the lowest bands for the pair of $ff = 0.4$ and 0.7 (up and down, respectively) and Fig. 2(c, d) –for 0.6, 0.8, and Fig. 2(e, f) for the system with included dipolar interaction: ff=0.6 and 0.9. Black lines marks the profiles for $k = 0$, and green lines – for $k = \pi/a$. Light blue and yellow colors denotes the regions in which the Py and Co strips are placed, respectively (see Fig. 1 in the manuscript). The Zak phase can be deduced by analyzing symmetry of the profiles and the edges of each band, i.e., for $k = 0$ and $k = \pi/a$.

References

1. Krawczyk, M., Sokolovskyy, M. L., Kłos, J. W. & Mamica, S. On the formulation of the exchange field in the Landau-Lifshitz equation for spin-wave calculation in magnonic crystals. *Adv. Condens. Matter Phys.* **2012**, 14, DOI: 10.1155/2012/764783 (2012).
2. Kaczér, J. & Murtinová, L. On the demagnetizing energy of periodic magnetic distributions. *Phys. Status Solidi (a)* **23**, 79–86, DOI: https://doi.org/10.1002/pssa.2210230108 (1974).
3. Zak, J. Symmetry criterion for surface states in solids. *Phys. Rev. B* **32**, 2218–2226, DOI: 10.1103/PhysRevB.32.2218 (1985).
4. Kohn, W. Analytic properties of Bloch waves and wannier functions. *Phys. Rev.* **115**, 809–821, DOI: 10.1103/PhysRev.115.809 (1959).
5. Zak, J. Berry's phase for energy bands in solids. *Phys. Rev. Lett.* **62**, 2747–2750, DOI: 10.1103/PhysRevLett.62.2747 (1989).
6. Xiao, M., Zhang, Z. Q. & Chan, C. T. Surface impedance and bulk band geometric phases in one-dimensional systems. *Phys. Rev. X* **4**, 021017, DOI: 10.1103/PhysRevX.4.021017 (2014).

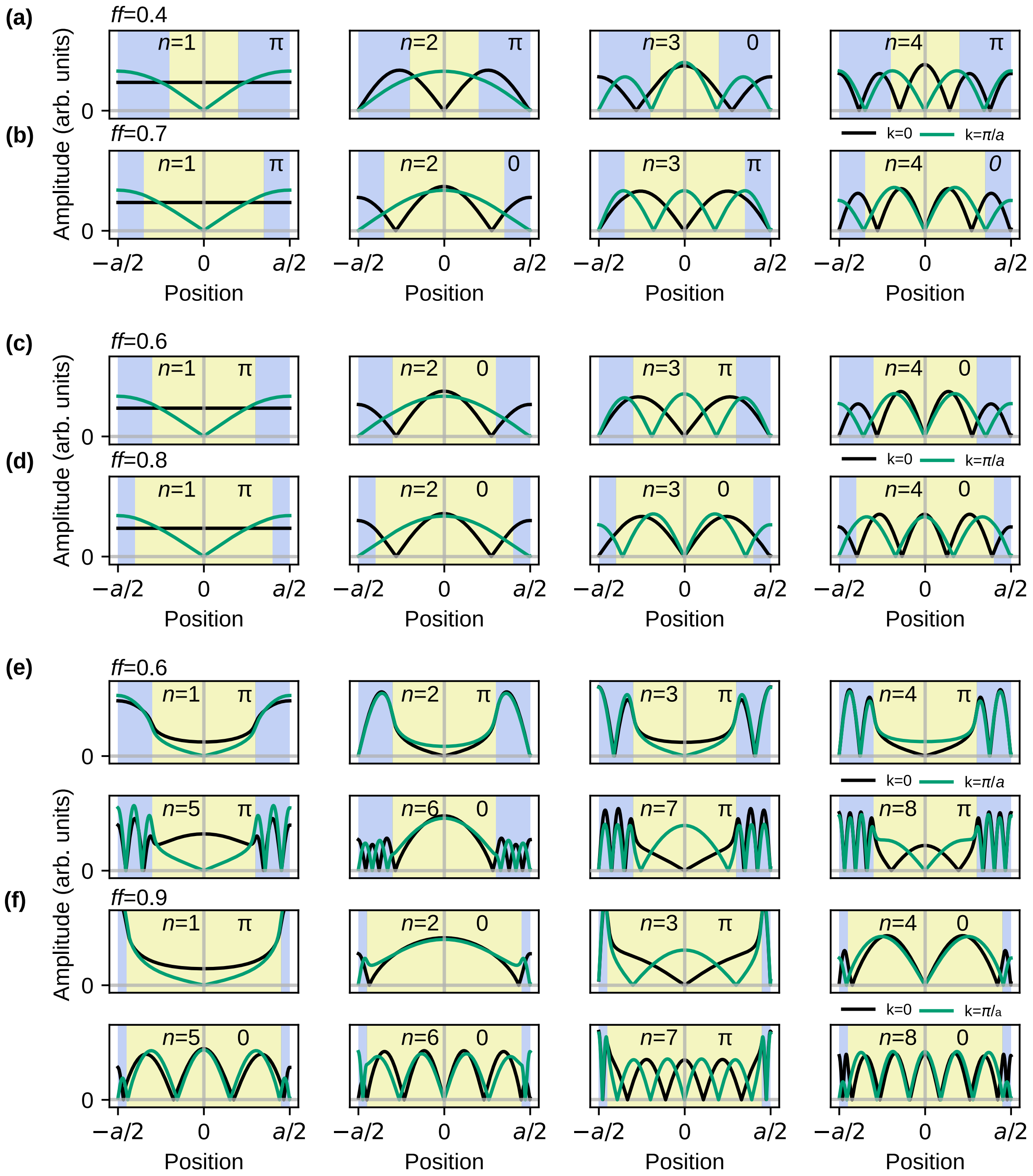

Figure 2. The SW's profiles $|m_\perp|$ within the unit cell at the edges of BZ. Black line is calculated for $k = 0$, green line for $k = \pi/a$. The values of the SW profiles and their derivatives allow determining the Zak phase for the band. (a) Results for $ff = 0.4$. For $n = 1$, $n = 2$ and $n = 4$ modes have different derivatives in the middle of unit cells, that suggest value of Zak phase equal to π. For $n = 3$ Zak phase is equal to 0. (b) Results for $ff = 0.7$. For $n = 1$ and $n = 3$ Zak phase is equal π, and for $n = 2$ and $n = 4$ Zak phase is equal 0. (c) Results for $ff = 0.6$. For $n = 1$ and $n = 3$ Zak phase equal to π. For $n = 2$ and $n = 4$ Zak phase is equal to 0. (d) Results for $ff = 0.8$. Only for $n = 1$ Zak phase is equal π, everywhere else is equal to 0. For exchange-dipolar waves, 8 SW's profiles are presented. (e) Results for $ff = 0.6$. For $n = 1$, $n = 2$, $n = 3$, $n = 4$, $n = 5$, $n = 8$ Zak phase is equal to π, everywhere else is equal to 0. (f) Results for $ff = 0.9$. For $n = 1$, $n = 3$, $n = 7$ Zak phase is equal to π, everywhere else is equal to 0.
